# Supplementary material for: Clinical Pattern of Preoperative Positron Emission Tomography/Computed Tomography (PET/CT) Can Predict the Aggressive Behavior of Resected Solid Pseudopapillary Neoplasm of the Pancreas
Source: Cancers (Basel). 2021 Apr 27;13(9):2119. doi: 10.3390/cancers13092119 (PMC8125428; doi:10.3390/cancers13092119)
Supplement: Supplementary file 1 [file cancers-13-02119-s001.zip › cancers-1200564-supplementary.pdf]

cancers

Supplementary Materials: Clinical Pattern of Preoperative Positron Emission Tomography/Computed Tomography (PET/CT) Can Predict the Aggressive Behavior of Resected Solid Pseudopapillary Neoplasm of the Pancreas

Ji-Su Kim, Emmanuel II-Uy Hao, Seoung-Yoon Rho, Ho-Kyoung Hwang, Woo-Jung Lee, Dong-Sub Yoon and Chang-Moo Kang

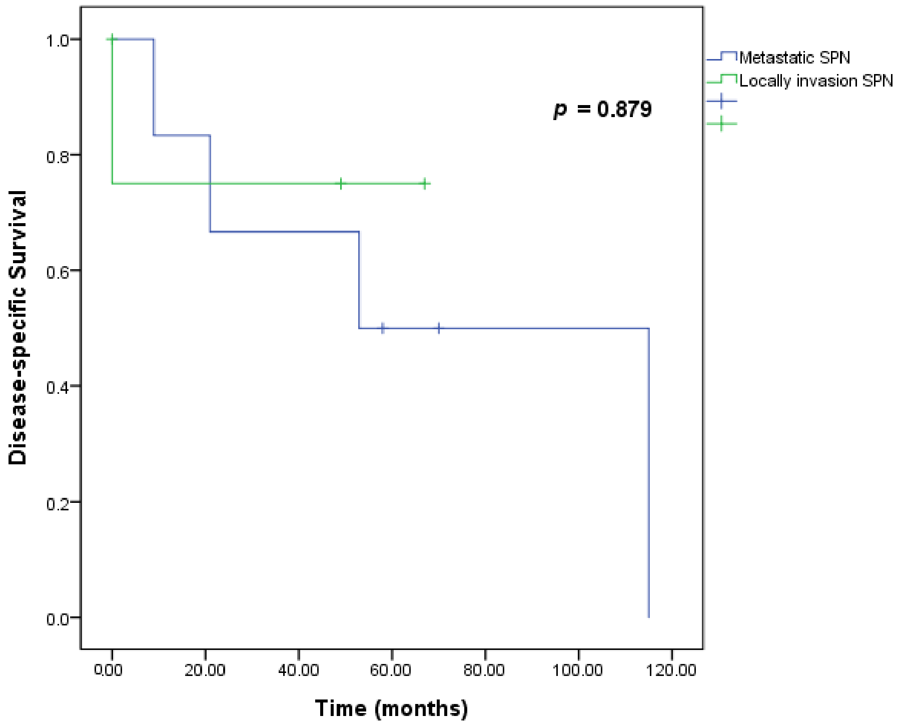

Figure S1. Disease-specific Survival plot for Aggressive versus Non-aggressive SPN; SPN, Solid pseudopapillary neoplasm.

Table S1. Characteristics of patients with metastatic and Locally invasion solid pseudopapillary tumor.

Variables	Metastatic SPN (*n* = 6)	Locally Invasion SPN (*n* = 4)	*p*-value
Age	-	-	-
≥40*	5 (83.3%)	2 (50%)	0.500
19–39	0	1 (25%)	>0.999
≤18	1 (16.7%)	1 (25%)	>0.999
Sex (F/M)	5/1	2/2	0.500
BMI	21.2 ± 4.45	22.5 ± 5.74	0.710
Symptomatic (yes/no)	5/1	4/0	>0.999
Location (proximal/distal)	1/5	3/1	0.190
Tumor size	-	-	-
≥10 cm	3 (50%)	1 (25%)	0.571
≥5 cm	3 (50%)	3 (75%)	0.571
≥2 cm	5 (83.3%)	4 (100%)	>0.999
PET configuration*	-	-	-
Type III	3 (60%)	4 (100%)	>0.999
Non-type III	2 (40%)	0	>0.999
Complication (yes/no)	1/5	3/1	0.190
Microscopic pathology	-	-	-
Margin	All negative	All negative	-
Capsular invasion	1 (16.7%)	1 (25%)	>0.999
Lymphovascular invasion	2 (33.3%)	1 (25%)	>0.999
Perineural invasion	2 (33.3%)	1 (25%)	>0.999
Ki-67	4.63 ± 5.31	1.00 ± 0.0	0.266

Values are *n* (%), mean ± standard deviation. PET, positron emission tomography; BMI, body mass index; WBC, white blood cell; CI, confidence interval.
